# Perceptions of opioid use and impact on quality of life in patients with musculoskeletal conditions within online health community forums

**DOI:** 10.1093/rap/rkab078

**Published:** 2021-11-10

**Authors:** Hassan Rana, Goran Nenadic, William G Dixon, Meghna Jani

**Affiliations:** 1 The University of Manchester Medical School; 2 Centre for Epidemiology Versus Arthritis, Centre for Musculoskeletal Research, University of Manchester; 3 Department of Computer Science, University of Manchester, Manchester; 4 Rheumatology Department, Salford Royal NHS Foundation Trust, Salford, UK

**Key message**

- Rich narratives of opioid-prescribed patients highlighted under-recognized themes associated with drug safety, including adverse drug reactions not frequently discussed, and their impact on quality of life.

Dear Editor, Opioids for chronic pain have received considerable notoriety, especially owing to an ‘opioid epidemic’ affecting North America and several Western countries [1, 2]. Qualitative work about patient perceptions through interviews has identified unintended consequences of this epidemic, including increased anxiety about being prescribed opioids in Canada [3]. Across Europe, research evaluating the patient perspective of prescribed opioid use is scarce. This was highlighted by a recent Public Health England report, reporting limited understanding of patients’ experiences of prescription opioids, which can include physical, emotional and social side effects [4].

Social media provides a valuable platform for patients living with disease to discuss their positive/negative medication experiences, holistic impact and to seek peer support/advice. Such patient perspectives may not always be communicated to health-care professionals or coded in electronic health records. Additionally, these data can complement traditional methods of drug safety, particularly for signal detection of rare events, niche areas such as drug abuse or evaluating the impact on quality of life (QOL) even of medically non-serious adverse drug reactions (ADRs) [5, 6]. An example of such a platform is HealthUnlocked, Europe’s largest social network for health, with >1.4 million members in 500 patient communities covering >250 conditions. With musculoskeletal conditions being one of the most common indications for prescribing opioids, the aim of this study was to evaluate perceptions and key themes from discussions within musculoskeletal communities on the HealthUnlocked platform. University ethics approval was obtained (UREC reference: 2019-7922-11765).

HealthUnlocked posts have no character limit, which allows unique opportunities for more detailed patient accounts. Of the 8021 posts on HealthUnlocked between 1 September 2015 and 1 September 2019, 7481 (93%) posts were from opioid users who provided consent for research. The date range was chosen for data availability and being before 2020 to avoid potential impact of the coronavirus disease 2019 pandemic on the results. Of the 7481 posts, 574 posts were from musculoskeletal-specific forums (RA, lupus, SSc, PMR/GCA, OA, FM and joint hypermobility), then de-identified by HealthUnlocked for research. Where geolocation was available, the majority of posts were from the UK. The average (mean) word count was 202 words per post and ranged between 8 and 1655 words. A manual thematic analysis was performed on a sample of 100 randomly selected posts, independently by two authors (H.R. and M.J.). To evaluate the impact on QOL, where described, themes associated with opioids were mapped to domains and subdomains (facets) of a QOL tool using all posts. The choice of which health-related QOL instrument to use was made by comparing multiple instruments, including musculoskeletal-specific QOL tools, by mapping a small number of posts with mention of a medication to each domain/facet (using a different HealthUnlocked dataset). WHO-QOL, developed by the World Health Organization, was found to be the best tool to capture the spectrum of QOL issues in this context. It yields a multi-dimensional profile across six domains, namely physical, psychological, level of independence, social relationships, environment and spirituality/religious beliefs [7].

Thematic analysis revealed key opioid-related themes related to ADRs (*n* = 45), ineffectiveness (*n* = 29) and opioid alternatives (*n* = 8), including using cannabidiol (CBD) oil, turmeric, probiotics and kratom. Less common themes were opioid withdrawal (*n* = 5), buying illicit opioids to alleviate chronic pain (*n* = 3) and effectiveness (*n* = 3). The ADRs most discussed were cognition-related effects, including brain fog (*n* = 8) and confusion (*n* = 4); gastrointestinal effects, such as nausea/vomiting (*n* = 11) and constipation (*n* = 4); and effect on sleep, including both insomnia (*n* = 7) and somnolence (*n* = 3). Also reported were headaches (*n* = 5), dizziness (*n* = 3) and rarer ADRs, including itching, sweating, seizures, memory loss, poor concentration and irritability. The presence of ADRs was accompanied by rich narratives highlighted in Table  1 (e.g. ‘Tonight, I have hit the Tramadol, so off to wacky land I go again’). Discussions about ADRs focused on several subthemes include their nature, probability of ADRs, impact on QOL, balancing harms with benefits, frustrations about the lack of information provided regarding ADRs and their addictive potential (Table  1).

**Table 1:** Thematic analysis of discussions about opioids on HealthUnlocked

Themes	Type of adverse drug reaction	Example of Patient quote^a^
1. Adverse drug reactions		
1a. Nature	Brain fog	Took tramadol as prescribed to help with pain. But they leave me feeling drugged up and dopey.
		I have a high intolerance to opiates. They turn me into a lethargic zombie who can’t string two words together.
	Headaches	I recently started a new opiate. Last night I had 1 h sleep and woke up with the worst inner tremors inside my head. It felt like a drill was going inside my head. I put my tongue in between my teeth, and it stopped for a while.
	Fatigue	I was started on buprenorphine and codeine, but should I feel SO tired all the time? I really don’t want to get up in the morning, I feel so awful.
	Constipation	Having really bad constipation on co-codamol. It wasn’t so bad at first, but now I’m getting cramps and bloating too.
	Vomiting	My doctor prescribed 25 μg fentanyl patches, and I applied one last night as per directions. This morning was violently vomiting. It didn’t settle and was unable to tolerate even sips of water. I was so concerned about becoming dehydrated I took the patch off.
1b. Rare effects	Seizure	After taking tramadol I had a seizure resulting in being hospitalized and dislocating my shoulder (never had a seizure before). Looking into it myself I realized the tramadol was causing the seizures. When I confronted my doctor, he said he had never heard of this happening before, although it’s well documented on the Web.
	Insomnia and sensory disturbance	Had my pain meds changed to tramadol. Here I am again early hours of the morning after 2 h sleep. I’m going crazy. The final straw is the creeping feeling that I get in my legs. I’m pacing the downstairs of the house trying to get rid of the sensations. Can anyone please help?
	Irritability	I have tramadol prescribed, but if I take it I become snappy and quick tempered. I stopped taking it for my wife’s benefit. Same thing for co-codamol.
1c. Probability	Itching	How commonly do people experience severe itching on opioids that also stings all over their body? I have had this now for 14 months, and my GP thinks this is the tramadol.
1d. Impact on quality of life	Insomnia	Taking tramadol has only given me side effects. I can’t sleep. I can’t type for any length of time and I love writing. I can’t concentrate long enough to read. My libido has disappeared with my energy.
	Effect on concentration	
	Reduced libido	
	Fatigue	I take the (opioid) drug then wait. But I feel so beyond tired that there are no words. Coffee or sleep, nothing helps. I can’t function well at work and stopped working a few months ago. I’m not angry; I feel emotionless.
	Effect on mood	
1e. Balancing harms and benefits	Confusion	I didn’t sleep well last night because of my shoulder pain. Tonight, I have hit the tramadol, so off to wacky land I go again. On the plus side, I can move my shoulder more.
	Brain fog	It gives me slightly better sleep, although I wake five or six times and get up feeling like I have the hangover from hell.
1f. Lack of awareness about information on adverse effects/addiction potential	Addiction	The doctor gave me dihydrocodeine for 10 years for pain, which gave me the worst addiction you could imagine. After an overdose episode, I asked him about the length of time he prescribed this evil drug.
		Still in state of shock, anger and very upset. Not once has any side effects nor dependency symptoms been brought to my attention. I personally feel doctors have responsibility to opioid users.
	Low blood pressure	I found out that my codeine could be causing my low blood pressure. Has anyone else been told about this or heard anything about it?
2. Ineffectiveness	Memory loss	I tried opioids. It doesn’t help my pain and I feel like I am having memory loss.
		Have very bad pain in neck and shoulder. Was given co-codamol and it hasn’t done a thing.
3. Withdrawal from opioids		I stopped taking tramadol that I've been on for a year. I think it makes me tremble and shake inside and out; the heat is really making me feel ill. I’ve had severe diarrhoea over the weekend, but no one else I live with has had this. I’m worried it’s all because of stopping the tramadol and am desperate for any advice please!
4. Discussing alternative treatments		I’ve got some CBD oil and have started to taper off my opioids. I’m hoping the CBD oil will help with the pain instead, even if I have to pay for it. It’s better than the damage I can do with these meds.
5. Buying illicit opioids for chronic pain		I have been on opioids for >10 years and I decided they were no longer helping my pain. I found myself buying them off the street because I was sick of being in pain. I then woke up one morning and said enough is enough, I’m fed up with being addicted to these pills.
6. Effectiveness		Recently tried tramadol and it has helped take the edge off the pain.

a Quotes have been paraphrased and abbreviated from HealthUnlocked while preserving the message to protect patient anonymity. Themes have been listed in order of frequency.

CBD: cannabidiol; GP: general practitioner.

Effects on QOL most frequently mapped to the WHO-QOL physical domain, which included facets of pain/discomfort, energy/fatigue and sleep/rest (*n* = 53). This was followed by impact on the psychological domain (*n* = 19; thinking, memory/concentration, self-esteem and negative feelings facets) and the level of independence domain (*n* = 10), with impact on activities of daily living and work capacity. Less commonly discussed impacts were on the social relationships domain, with influence on personal relationships and sexual activity (*n* = 4), and the environment domain that includes physical safety and financial resources (*n* = 4).

Patient narratives can reveal important and under-recognized themes that may not be available through traditional methods of pharmacovigilance and pharmacoepidemiology. Our analysis revealed that ADRs infrequently discussed in clinics, such as brain fog/confusion, withdrawal and addictive potential of prescribed opioids in musculoskeletal patients, can have considerable impact on patients’ QOL. Patient-generated data have the ability to provide rich information using social media and mobile health, through exploration of the nature, probability and importance of ADRs to the person experiencing them [8]. In the future, automated text-mining using natural language processing to perform topic modelling and sentiment analysis on larger datasets could help to determine emergent themes that provide valuable insights about patient experiences. 

*Funding:* The work was supported by the Centre for Epidemiology Versus Arthritis (Reference: 20380) and Healtex, the UK Healthcare text analytics research network (which is funded through EPSRC). M.J. is funded by a National Institute for Health and Research (NIHR) Advanced Fellowship (NIHR301413) for this research project. The views expressed are those of the author(s) and not necessarily those of the NIHR or the Department of Health and Social Care. 

*Disclosure statement:* W.G.D. has received consultancy fees from Google and Bayer, unrelated to this work. M.J. is a member of the The Medicines and Healthcare products Regulatory Agency (MHRA) Opioid Expert Working group. The remaining authors have declared no conflicts of interest.

## Data availability statement

Anonymized data are available via HealthUnlocked for a fee and will be subject to ethics approval and contractual agreements. The dataset used for this analysis cannot be shared with third parties due to contractual agreements and information governance issues.
